# Comparative analysis of teratogen-induced malformations and gene expression across zebrafish strains in early development

**DOI:** 10.1016/j.toxrep.2025.102117

**Published:** 2025-08-21

**Authors:** Chitose Taya, Kota Ujibe, Shinnosuke Shimodaira, Aoto Sakamoto, Seiji Wada, Makoto Kashima, Hiromi Hirata

**Affiliations:** a Department of Chemistry and Biological Science, College of Science and Engineering, Aoyama Gakuin University, Sagamihara 252-5258, Japan; b Faculty of Science, Toho University, Funabashi 274-8510, Japan

**Keywords:** Malformation, Mortality, Strain, Teratogen, Toxicology, Zebrafish

## Abstract

Zebrafish embryos are widely used in developmental toxicity testing. However, the extent to which genetic background influences susceptibility to teratogenic compounds remains incompletely understood. We here evaluated inter-strain variability in both phenotypic and transcriptomic responses to six model teratogens using five commonly utilized zebrafish strains, AB, TU, RW, WIK, and PET. All test compounds, valproic acid, hydroxyurea, methotrexate, acitretin, topiramate, and ibuprofen, elicited concentration-dependent developmental toxicity characterized by malformations at moderate doses and lethality at higher concentrations. Despite distinct toxicodynamic profiles, the incidence and severity of phenotypic outcomes were highly consistent across strains. Transcriptomic analysis was performed following exposure to valproic acid, hydroxyurea, and warfarin, revealing strong, dose-dependent gene expression changes that were largely conserved among strains. Principal component analysis demonstrated that chemical concentration, rather than strain, was the dominant driver of transcriptional variation. Minor strain-specific differences were observed at baseline or low-dose levels but did not alter the overall direction or magnitude of response. These findings demonstrate that zebrafish embryos from diverse genetic backgrounds exhibit broadly conserved developmental and molecular responses to teratogens. The minimal inter-strain variability supports the use of any wild-type strain, transgenic line, or even outbred population in developmental toxicity testing without compromising sensitivity or reproducibility. Our study reinforces the suitability of zebrafish as a robust vertebrate model in regulatory toxicology.

## Highlights

• Developmental toxicity was assessed using five common zebrafish wild-type strains.
• Six model teratogens caused consistent malformations and lethality across strains.
• Transcriptomic responses to teratogens were strongly dose-dependent and conserved.
• Strain had minimal impact on phenotypic or gene expression outcomes.
• Zebrafish embryos provide a robust and reproducible model for toxicity testing.

## Graphical Abstract

**Figure:**
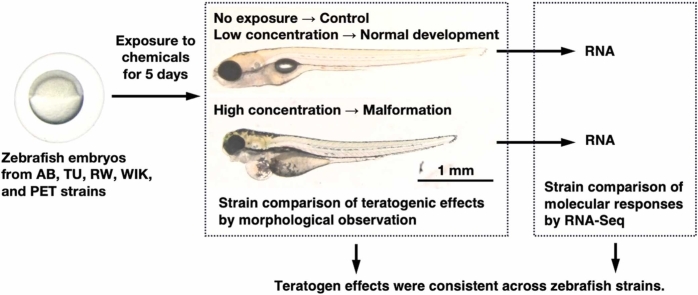

## 1 Introduction

Zebrafish (*Danio rerio*) has emerged as a powerful and versatile vertebrate model organism for a wide range of biological research, including developmental biology, genetics, neuroscience and drug discovery [8]. Its small size, rapid ex vivo development, early transparency, high fecundity, and genetic tractability make it particularly well-suited for studying early developmental processes and deficiency [13], [30]. Furthermore, the significant genetic conservation between zebrafish and humans, estimated at approximately 70 % for protein-coding genes, enhancing its translational relevance for biomedical research [1], [10].

Over several decades of research, numerous zebrafish strains have been established in laboratories and are now widely used in the world [37]. Commonly used wild-type laboratory strains include AB, Tübingen (TU), RW, and WIK, each originating from different local distributors or geographical regions [4], [22], [26], [27], [30]. The PET strain, which is available from a local distributor, is a long-term pet store stock not subjected to the same controlled breeding practices as laboratory-maintained strains. Recent whole genome sequencing and phylogenetic analysis confirmed that these strains share a common ancestor, with the WIK strain forming a distinct subgroup from the others [27]. Genetic drift and selective breeding pressures during domestication have led to the accumulation of genetic polymorphisms in each strain, potentially resulting in phenotypic divergence among zebrafish strains [9], [27]. Indeed, several studies have reported subtle yet notable inter-strain differences in various aspects of zebrafish biology. For instance, morphological variations in growth and body size among strains have been documented [18], [19]. At the physiological level, strain-specific differences have been observed in larval hemostasis, adult stress responses, and reproductive traits [3], [6], [32]. Furthermore, behavioral differences, such as locomotion, circadian rhythms, and shoaling, have also been identified in both larvae and adults across strains [5], [14], [17], [32], [33], [34].

During early embryonic development in zebrafish, a complex and tightly regulated cascade of molecular and cellular events orchestrates the formation of tissues and organs [23]. This intricate process is highly sensitive to environmental perturbations, including exposure to chemical compounds. Developmental toxicity, characterized by the induction of malformations, growth retardation, or embryonic lethality, can arise from the disruption of these critical developmental pathways [25]. Given the genetic diversity among zebrafish strains, it is plausible that these variations could influence their susceptibility to developmental toxicants. Previous investigations have also explored potential strain-specific differences in developmental toxicity. For example, studies have reported variations in sensitivity to certain chemicals based on the zebrafish strain used [7], [15], [16], [24]. These differences could be attributed to variations in xenobiotic metabolism, target protein affinity, or the efficiency of DNA repair mechanisms across different genetic backgrounds. Understanding the extent of inter-strain variability in developmental toxicity is crucial for ensuring the reproducibility and generalizability of toxicological studies using zebrafish as a model. However, most previous studies have been limited to either phenotypic endpoints or a narrow selection of strains, and few have integrated standardized malformation and embryo-fetal lethality (MEFL) assays with transcriptomic profiling across multiple genetically distinct zebrafish strains.

This study aims to comprehensively evaluate inter-strain variability in chemical susceptibility during zebrafish teratogenicity testing. We employed the MEFL assay, which is a standardized method for assessing developmental toxicity, across five commonly used zebrafish strains (AB, TU, RW, WIK, and PET), using a panel of six model teratogens administered at therapeutically relevant low doses. Furthermore, to investigate the underlying molecular mechanisms, we conducted RNA sequencing (RNA-Seq) to examine transcriptional responses in a subset of strains exposed to selected compounds at both low and high concentrations. By comparing phenotypic variability with transcriptomic differences, we conclude that genetic background has a limited influence on susceptibility to developmental toxicity in zebrafish. The combination of MEFL testing with RNA-Seq across multiple strains not only extends previous work but also has practical regulatory implications, as it supports the reproducibility of zebrafish-based developmental toxicity testing and could facilitate the refinement or broader adoption of internationally harmonized guidelines such as those of the OECD.

## 2 Materials and methods

### 2.1 Ethics declarations

This study was approved by the Animal Care and Ethics Committee of Aoyama Gakuin University and carried out according to the Animal Research Reporting of In VIVO Experiments (ARRIVE) guidelines and relevant regulations.

### 2.2 Animals

Zebrafish (*Danio rerio*) AB, TU, and WIK strains were purchased from Zebrafish International Resource Center in the University of Oregon, USA. The RW strain was obtained from National BioResource Project (NBRP), Japan. In addition to commonly used laboratory strains, we included the PET strain, which was obtained from a local pet store in Japan (Charm, Oizumi, Gunma). This strain was chosen to capture a broader spectrum of genetic diversity, as PET populations are expected to have undergone different founder effects, breeding histories, and environmental exposures compared to established laboratory strains. Zebrafish were reared and maintained in 1.7 L tanks in a recirculating Meito System at 28 ± 1 °C under a 14 h light and 10 h dark photoperiod according to the standard protocol [36]. Zebrafish larvae were raised in 90 mm dishes until 5 days post-fertilization (dpf) and then placed in the recirculation system at 5 dpf. Larval fish were fed Gemma Micro ZF 75 and paramecia twice a day from 5 dpf to 1 month post-fertilization (mpf). Juvenile fish (1–3 mpf) were fed Gemma Micro ZF 75 and brine shrimp twice a day. Adult fish (3 mpf ∼) were fed Otohime B2 or Gemma Micro ZF 300 and brine shrimp twice a day.

### 2.3 Test compounds

Seven test compounds (valproic acid, hydroxyurea, methotrexate, acitretin, topiramate, ibuprofen, and warfarin) listed in the ICH Reference Compound List [11] were used in this study. The test compounds were dissolved in dimethyl sulfoxide (DMSO) or water to prepare the master solutions as described previously [21]. These master solutions were diluted using E3 medium (5 mM NaCl; 0.17 mM KCl; 0.33 mM CaCl_2_; 0.33 mM MgSO_4_) [36]. In the case where we used DMSO as a solvent, the final DMSO concentration was set at 0.5 % for zebrafish embryo exposure.

### 2.4 Chemical exposure of zebrafish embryos

To evaluate inter-strain variability in chemical susceptibility during zebrafish embryogenesis, we performed MEFL testing based on the OECD test guideline 236 (TG236), with minor modifications to suit our experimental design. In brief, adult female and male zebrafish that generated embryos in previous week were used for mating. A pair of adult fish of AB, TU, RW, WIK, and PET strains were housed overnight in a breeding tank with a divider prior to mating. The following morning, the divider was removed to initiate mating after the light of the fish room turned on. Embryos of AB, TU, RW, WIK, and PET strains were collected within 1–3 h post-fertilization (hpf) to ensure synchronized developmental timing and incubated in system water at 28.5 °C in 90 mm petri dishes. Embryos were microscopically examined at 4–6 hpf, and any showing spontaneous developmental abnormalities, such as irregular cleavage, abnormal yolk morphology, or delayed development, were excluded from testing. Healthy embryos were randomly assigned to experimental groups and transferred to 24-well plates at one embryo per well in system water. At 4–6 hpf, system water was replaced to chemical-containing E3 medium. In one assay, 10–20 individual zebrafish embryos were tested at each concentration. They were placed in a 28.5 °C incubator with no medium change for up to 5 dpf and subjected to the assessment of viability and morphological abnormality under a stereomicroscope as described previously [20], [21].

### 2.5 RNA-Seq

RNA extraction from zebrafish larvae and RNA purification were described previously [31]. The RNA concentration was determined using the Quantus Fluorometer with QuantiFluor RNA System Kit (Promega) according to the manufacturer’s manual. RNA was diluted to a final concentration of 2 ng/µl and used to prepare library for RNA-Seq. Library preparation was conducted according to the Lasy-Seq ver 1.1 method. In brief, reverse transcription was done using SuperScript IV reverse transcriptase (Thermo Fisher Scientific) and indexed reverse transcription primers. Following program was used for reverse transcription: 65 °C 10 min; 80 °C 15 min; 4 °C forever in ProFlex PCR System. Products of reverse transcription were pooled and then purified using an equal volume of AMpure XP beads (Beckman Coulter) according to the manufacturer’s manual. Second strand synthesis was conducted with RNase H (Enzymatics) and DNA polymerase I (Enzymatics) at 16 °C for 2 h. Synthetic double-strand DNA (dsDNA) were treated with RNase T1 (Thermo Fisher Scientific) at 37 °C for 5 min. Fragmentation was conducted using 5x WGS Fragmentation Mix (Enzymatics) at 32 °C for 6.5 min followed by heat inactivation at 65 °C for 30 min. Ligation was conducted using 5x Ligation Mix (Enzymatics) at 20 °C for 15 min. Library amplification was conducted using KAPA HiFi HotStart ReadyMix (KAPA BIOSYSTEMS) using the following program: 95 °C 5 min; 98 °C 20 s, 60 °C 15 s, 72 °C 40 s, 12 cycles; 72 °C 3 min; 4 °C forever in QuantStudio 5 Real-Time PCR Systems (Thermo Fisher Scientific). The library was subjected to electrophoresis in Bioanalyzer 2100 (Agilent Technologies) with the Agilent High Sensitivity DNA kit (Agilent Technologies). Library concentration was determined using Quantus Fluorometer (Promega) with the QuantiFluor ONE dsDNA System kit (Promega) and diluted to 4 ng/µl. Sequencing of 150-bp paired-end reads was conducted using HiSeq X Ten (Illumina). Methods for mapping of sequencing data and counts of transcripts were described previously [12]. The principal component analysis (PCA) was performed in R software using RunPCA and FindVariableFeatures functions with default parameters.

## 3 Results

### 3.1 MEFL testing

**Fig. 1:**
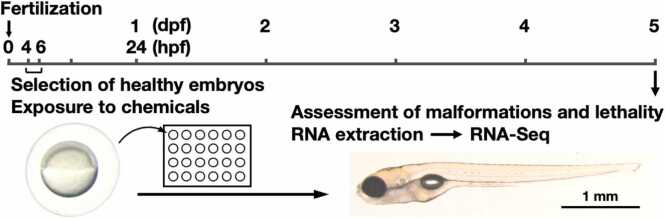
Schematic overview of the experimental design for the zebrafish MEFL test. Healthy zebrafish embryos were selected at 4–6 hpf for the MEFL testing. Immediately following selection, embryos were exposed to test chemicals in 24-well plates. The exposure continued under standardized conditions until 5 dpf, equivalent to 120 hpf. At the end of the exposure period, larvae were examined for morphological abnormalities and lethality. Representative larvae were then subjected to RNA extraction for transcriptomic profiling using RNA-Seq. This design enables the parallel assessment of developmental toxicity and gene expression changes in response to chemical exposure.

**Fig. 2:**
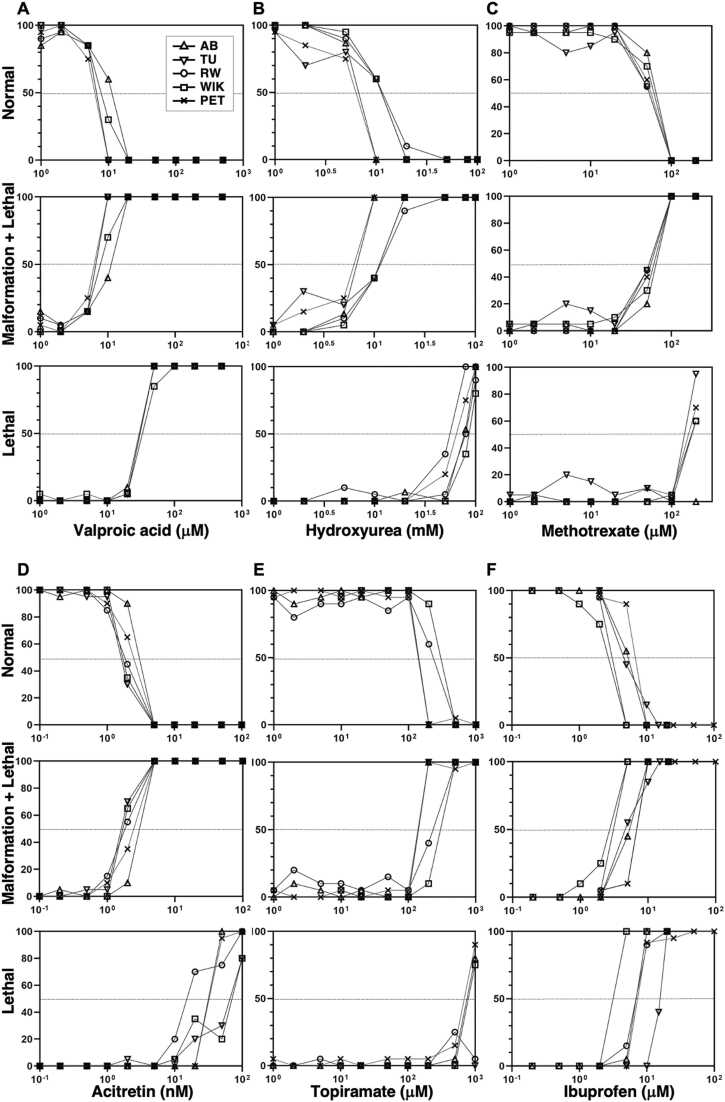
Comparable teratogenic and lethal responses were observed across five zebrafish strains upon chemical exposure. Each panel illustrates the dose-dependent effects of six representative compounds on zebrafish embryonic development assessed at 5 dpf. The data are shown for three outcome categories: percentage of embryos displaying a normal development (top row), the combined percentage of embryos exhibiting malformations or lethality (middle row), and embryos exhibiting lethality alone (bottom row). Embryos were exposed to the indicated chemicals from 5–6 hpf to 5 dpf. The x-axes represent the logarithmic concentration of each compound. The y-axes show the percentage of embryos falling within each category at each concentration tested. Zebrafish strains: AB (closed triangle), TU (open triangle), RW (circle), WIK (square), and PET (×). The six compounds tested include: (A) Valproic acid, (B) Hydroxyurea, (C) Methotrexate, (D) Acitretin, (E) Topiramate, and (F) Ibuprofen. The sample sizes (n) for each chemical concentration and zebrafish strain were shown in Supplementary Table S1. The number of biological replicates is one. Despite differences in genetic background, all strains exhibited consistent patterns of developmental toxicity and lethality, supporting the reproducibility of chemical responses across strains under standardized testing conditions.

To systematically investigate how genetic background influences developmental chemical sensitivity, we exposed embryos from five commonly used zebrafish strains, AB, TU, RW, WIK, and PET to a panel of six well-characterized model teratogenic compounds at the onset of 4–6 hpf (Fig. 1). These chemicals, valproic acid, hydroxyurea, methotrexate, acitretin, topiramate, and ibuprofen, were selected based on their known mechanisms of action and clinical relevance, as each has been associated with teratogenic effects in established animal models. All six test compounds elicited adverse developmental outcomes in a concentration-dependent manner at 5 dpf, although the nature and severity of the responses varied between chemicals. Valproic acid, an antiepileptic agent known to interfere with neural tube development, consistently induced a dose-responsive decline in the percentage of normal embryos in all strains. Moderate concentrations led to elevated rates of craniofacial and axial malformations, while higher doses resulted in significant lethality across the board (Fig. 2A). Hydroxyurea, which disrupts DNA synthesis, similarly caused malformations at intermediate concentrations and lethality at higher doses in all strain, suggesting only a subtle inter-strain variability in response thresholds (Fig. 2B). Methotrexate, a folate pathway inhibitor, triggered a robust and reproducible increase in both malformations and mortality with rising concentrations, and notably, the pattern of responses was nearly identical across the five strains, reflecting high reproducibility and minimal genetic variation in sensitivity to this compound (Fig. 2C). Acitretin, a synthetic retinoid, proved to be the most potent among the panel, producing pronounced teratogenic effects and embryo death even at nanomolar concentrations, indicative of high toxicity and low threshold for developmental disruption. This effect was uniformly observed in all strains, suggesting that the retinoid pathway is equally susceptible across different genetic backgrounds (Fig. 2D). In contrast, topiramate, an anticonvulsant drug, exhibited a higher toxicity threshold, with developmental abnormalities only emerging at concentrations of 100 μM or above, and lethality occurring at even higher levels. Importantly, the response patterns were consistent across all strains, and no apparent inter-strain differences in sensitivity were detected (Fig. 2E). Ibuprofen, a commonly used nonsteroidal anti-inflammatory drug (NSAID), produced dose-dependent effects characterized by malformations at lower micromolar concentrations and increased lethality at higher concentrations, again with consistent responses among all five strains tested (Fig. 2F). Collectively, these findings demonstrate that although each compound displays a distinct toxicity profile, the overall patterns of phenotypic responses were broadly conserved among the five zebrafish strains. This suggests that zebrafish embryos exhibit comparable developmental susceptibility to a range of chemical insults, reinforcing their utility as a robust vertebrate model for high-throughput toxicological screening.

### 3.2 Transcriptome analysis

To investigate the impact of chemical exposure on transcriptional responses during early zebrafish development, we conducted a comparative transcriptomic analysis across five zebrafish strains: AB, TU, RW, WIK, and PET. Embryos from each strain were exposed to three well-characterized model compounds, valproic acid, hydroxyurea, and warfarin, administered at increasing concentrations to capture both subtle and overt molecular responses. Exposure was carried out at the onset of 5–6 hpf and total RNA was extracted from individual larvae at 5 dpf to enable high-resolution transcriptional profiling. The resulting transcriptomic datasets were subjected to PCA to visualize global gene expression variation and identify patterns associated with both treatment concentration and genetic background. This approach allowed us to examine whether strain-specific differences contribute significantly to molecular responses or whether chemical exposure is the dominant factor influencing gene expression profiles. The PCA plots revealed clear, concentration-dependent shifts in transcriptional signatures across all three compounds tested.

**Fig. 3:**
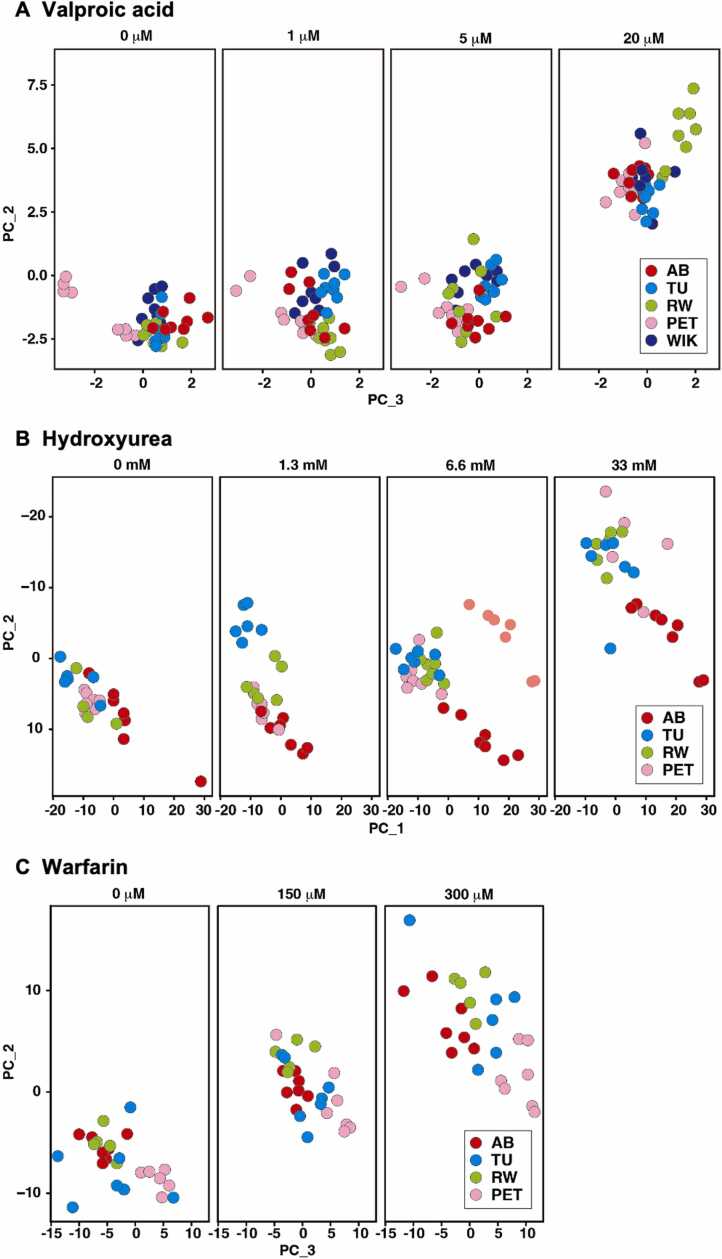
Comparable transcriptomic responses to teratogens across multiple zebrafish strains. The PCA of RNA-Seq data from whole larvae at 5 dpf exposed to increasing concentrations of (A) valproic acid, (B) hydroxyurea, and (C) warfarin. Each dot represents a single larva from one of five strains: AB (red), TU (blue), RW (green), PET (pink), and WIK (purple). The sample sizes (n) for each chemical concentration and zebrafish strain were shown in Supplementary Table S2. The number of biological replicates is one. Note that dose-dependent shifts in gene expression patterns were observed consistently across all strains, indicating similar transcriptional responses to chemical exposure.

For valproic acid, which is known to affect histone deacetylation and neural development, embryos exposed to the highest dose (20 µM) clustered distinctly from those exposed to lower doses and from untreated controls (Fig. 3A). This separation was consistent across all five strains, indicating a robust and reproducible transcriptional response to valproic acid at higher concentrations. Interestingly, even at the control and low-dose levels, the PET strain exhibited a slight displacement in PCA space compared to the other strains, suggesting potential baseline differences in gene expression or early-stage responses unique to this strain. Similarly, hydroxyurea, a ribonucleotide reductase inhibitor that interferes with DNA synthesis, induced significant transcriptional alterations at elevated concentrations (Fig. 3B). Embryos treated with higher doses formed well-defined clusters distinct from those of lower-dose or control groups in the PCA space, emphasizing a clear dose-response relationship. In this case, the AB strain plots were marginally separated from the other strains, pointing to possible subtle differences in the molecular response threshold, although the overall transcriptional direction remained consistent. Warfarin, a vitamin K antagonist widely used as an anticoagulant, also elicited strong, dose-dependent transcriptional changes (Fig. 3C). The PCA revealed that embryos exposed to 300 µM of warfarin grouped separately from those at lower concentrations and controls. This pattern mirrors the transcriptional shifts observed with the other two compounds, further validating the sensitivity and specificity of the approach. Despite these pronounced dose-dependent changes in gene expression, embryos from different strains generally clustered closely together under equivalent exposure conditions. This suggests that the chemical treatments exerted a stronger influence on transcriptional profiles than genetic background alone. The degree of inter-strain variability was relatively small and did not overshadow the compound-induced effects, indicating that zebrafish embryos from different strains mount broadly conserved molecular responses to teratogens. The transcripts that showed downregulation or upregulation after exposure to varying concentrations of valproic acid, hydroxyurea, or warfarin were listed in Supplementary Tables S3–S18.

Taken together, these findings demonstrate that although minor strain-specific transcriptional nuances exist, particularly at baseline or low exposure levels, the overall gene expression responses to chemical insults are largely consistent across diverse genetic backgrounds. This supports the validity of using multiple zebrafish strains in toxicogenomic studies and highlights the robustness of zebrafish embryos as a model for assessing teratogenic mechanisms at the transcriptomic level.

## 4 Discussion

### 4.1 Limited inter-strain variability in teratogenicity

Genetic background can influence chemical susceptibility in animal models, as demonstrated in rodents and, to a lesser extent, in previous zebrafish studies [3], [5], [6], [14], [17], [18], [19], [29], [32], [33], [34]. Our recent phylogenetic analysis of 13 zebrafish strains revealed that all strains share a common ancestor, but the WIK strain is distinctly separated, forming a unique subgroup. Based on this, we initially hypothesized that WIK embryos might exhibit differential responses to chemical exposures compared to embryos of other strains. However, our MEFL results indicate that strain-related variability was minimal in any strain under the exposure conditions tested. Notably, all six compounds, valproic acid, hydroxyurea, methotrexate, acitretin, topiramate, and ibuprofen, elicited comparable, dose-dependent developmental toxicity characterized by increased malformations at intermediate doses and embryo lethality at higher concentrations. These phenotypic outcomes were consistent in both incidence and severity across all strains, particularly at moderate to high exposure levels. The observed phenotypes aligned with the known mechanisms of action and established teratogenic profiles of these compounds in humans and other model organisms. For example, valproic acid, a histone deacetylase inhibitor, disrupted embryonic development in a dose-dependent manner across all strains, leading to lower jaw hypoplasia and cardiac malformations as reported in the previous zebrafish studies [2], [20], [21], [28], [35]. This is consistent with its reported developmental toxicity in humans, including craniofacial abnormalities, congenital heart defects, and neural tube defects. Similarly, hydroxyurea, which interferes with DNA replication, produced comparable developmental outcomes such as microcephaly, shortened fins, and cardiac malformations in all zebrafish strains tested, mirroring its known embryotoxicity in mammals [2], [20], [21], [28], [35].

### 4.2 Limited inter-strain variability in transcriptome

The transcriptome offers a molecular perspective on chemical susceptibility and enables the assessment of whether genetic background modulates the direction or extent of gene expression changes during toxicant exposure. While MEFL testing highlights overt outcomes such as malformation at moderate doses and lethality at higher doses, RNA-Seq reveals subtle internal responses even at low, non-hazardous concentrations. PCA of transcriptomic data demonstrated dose-dependent shifts in gene expression, progressing from control to high-exposure conditions. Embryos exposed to higher concentrations formed distinct clusters from those in control, low, or moderate exposure groups, reflecting robust transcriptional activation or repression. Crucially, PCA plots indicated that despite the strong dose effect, embryos from different strains clustered similarly under identical exposures. Some minor strain-specific variations were observed (e.g., PET with valproic acid, TU and PET with hydroxyurea), but these did not disrupt the overall clustering patterns or dose-response trends. These discrepancies likely stem from intrinsic expression baselines or early regulatory dynamics and do not impair the consistency or interpretability of transcriptional outcomes.

Taken together, our combined phenotypic and transcriptomic analyses show that zebrafish embryos from different genetic backgrounds respond consistently to teratogenic exposures, especially at moderate to high doses. This consistency supports the utility of zebrafish for regulatory toxicology, where reproducibility is essential. Future work should explore additional chemical classes and employ earlier time points and time-resolved transcriptomics to better capture initial molecular responses. Integration with proteomic and metabolomic data may further enhance the utility of zebrafish in predictive toxicology and human health risk assessment.

## 5 Conclusion

Our study was designed to systematically evaluate the extent of inter-strain variability in both phenotypic and molecular responses to teratogenic chemical exposure during zebrafish embryogenesis. By applying the MEFL assay and RNA-Seq, we revealed broadly consistent patterns of concentration-dependent malformations, lethality, and transcriptomic responses across all five AB, TU, RW, WIK, and PET strains, suggesting that despite genetic differences, zebrafish embryos exhibit largely conserved susceptibility to chemical exposure. The low inter-strain variability justifies the use of various wild-type and transgenic strains, including outbred populations, in developmental toxicity testing.

## CRediT authorship contribution statement

**Chitose Taya:** Investigation. **Kota Ujibe:** Investigation. **Hiromi Hirata:** Writing – review & editing, Writing – original draft, Validation, Supervision, Project administration, Methodology, Investigation, Funding acquisition, Conceptualization. **Seiji Wada:** Investigation. **Makoto Kashima:** Investigation. **Shinnosuke Shimodaira:** Investigation. **Aoto Sakamoto:** Investigation.

## Declaration of Competing Interest

The authors declare the following financial interests/personal relationships which may be considered as potential competing interests: Hiromi Hirata reports financial support was provided by Japan Agency for Medical Research and Development. Hiromi Hirata reports a relationship with Japan Chemical Industry Association that includes:. If there are other authors, they declare that they have no known competing financial interests or personal relationships that could have appeared to influence the work reported in this paper.
